# Supplementary material for: Overexpression of Sweet Potato Carotenoid Cleavage Dioxygenase 4 (IbCCD4) Decreased Salt Tolerance in Arabidopsis thaliana
Source: Int J Mol Sci. 2022 Sep 1;23(17):9963. doi: 10.3390/ijms23179963 (PMC9456075; doi:10.3390/ijms23179963)
Supplement: Supplementary file 1 [file ijms-23-09963-s001.zip › Caption of the supplementary materials.pdf]

Table S1 The primers used in this study.

Figure S1 The multiple sequence alignment of CCD4 proteins from 16 plant species.

GenBank accession numbers of the CCD4 proteins include IbCCD4 (*Ipomoea batatas,*), ItCCD4 (*Ipomoea triloba*, XP_031124907.1), InCCD4 (*Ipomoea nil*, XP_019156361.1), StCCD4 (*Solanum tuberosum*, XP_006359966.1), SlCCD4 (*Solanum lycopersicum*, XP_004246004.1), VvCCD4 (*Vitis vinifera* , AGT63321.1), OfCCD4 (*Osmanthus fragrans* , ABY60887.1), DmCCD4 (*Dendranthema morifolium,* BAF36656.2), PpCCD4 (*Prunus persica*, PRUPE_1G255500), AtCCD4 (*Arabidopsis thaliana*, AT4G19170), MdCCD4c (*Malus domestica,* XP_008340019.2), ZmCCD4 (*Zea mays,* PWZ28009.1)*,* CitCCD4 (*Citrus*, AB781691), CitCCD4b (*Citrus clementina,* ABC26012), CsCCD4b (*Crocus sativus*, ACD62477), and TaCCD4 *(Triticum aestivum,* QEX50885.1). Triangles indicate the four highly conserved histidine residues as an iron-ligating cofactor; asterisks indicate the conserved glutamates or aspartate for fixing the iron-ligating histidine residues.
