# Supplementary material for: Overexpression of Sweet Potato Carotenoid Cleavage Dioxygenase 4 (IbCCD4) Decreased Salt Tolerance in Arabidopsis thaliana
Source: Int J Mol Sci. 2022 Sep 1;23(17):9963. doi: 10.3390/ijms23179963 (PMC9456075; doi:10.3390/ijms23179963)

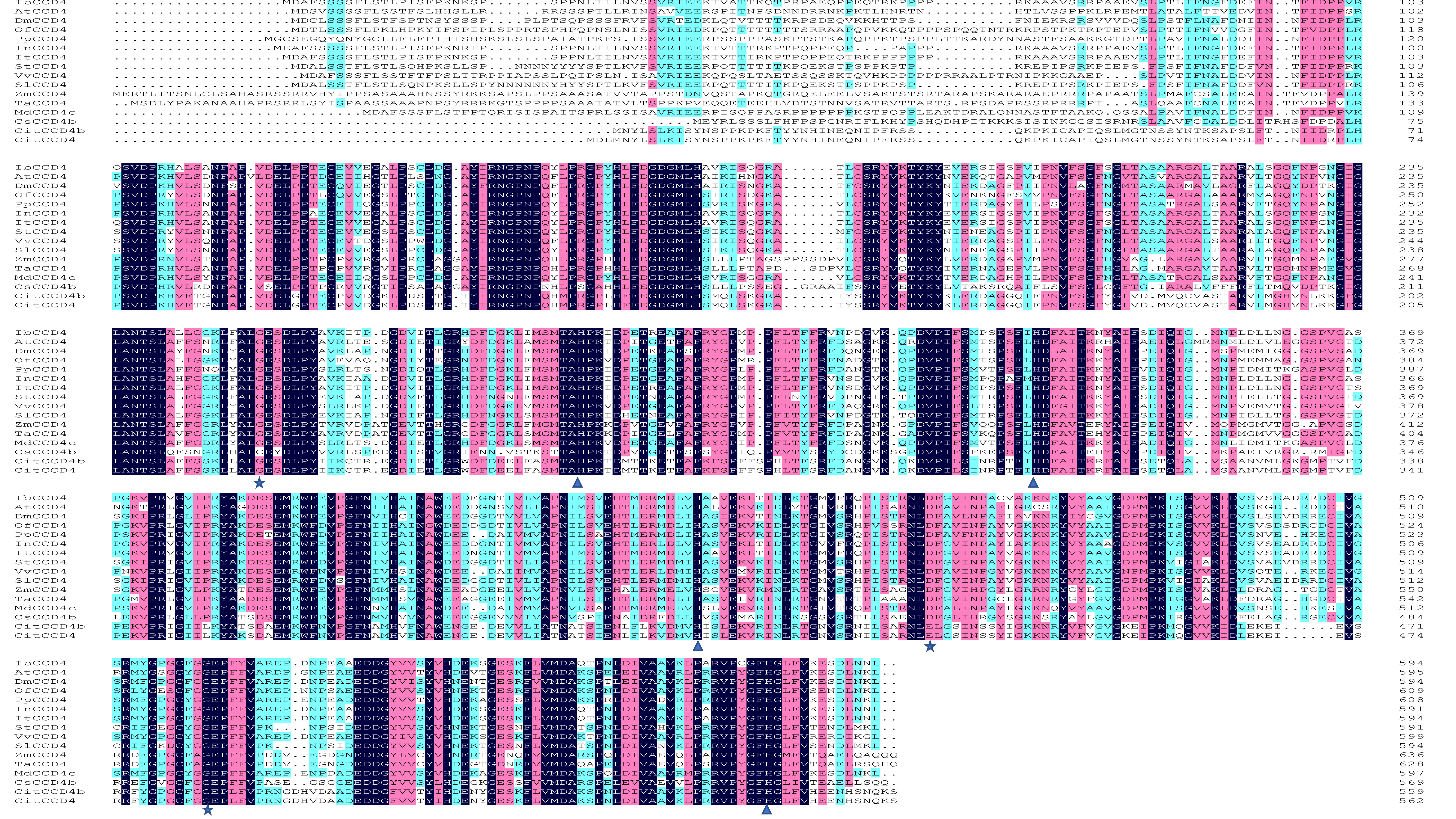

Supplement: Supplementary file 1 [file ijms-23-09963-s001.zip › Figure S1.pdf]
